# Horses Failed to Learn from Humans by Observation

**DOI:** 10.3390/ani10020221

**Published:** 2020-01-29

**Authors:** Maria Vilain Rørvang, Tina Bach Nielsen, Janne Winther Christensen

**Affiliations:** 1 Swedish University of Agricultural Sciences, Dept. Biosystems and Technology, 23053 Alnarp, Sweden; 2 Aarhus University, Dept. Animal Science, 8830 Tjele, Denmark

**Keywords:** horse, cognition, social learning, welfare, training, social transmission, inter-species

## Abstract

**Simple Summary:**

The behavior of animals can be altered in various ways. Horses interact on an everyday basis with humans, and some studies suggest that horses can learn new behavior from observing humans. However, scientific findings are conflicting. This study seeks to investigate if horses can learn to solve an instrumental task of opening a box, by observing human demonstration. We control for social transmission mechanisms, which require lower cognitive complexity than actual social learning. One human demonstrator either (A) fully demonstrated how to solve the task, (B) partially demonstrated the task, or (C) provided no demonstration. Thirty horses were randomly assigned to one of these treatments, and their success and behavior was observed. Horses watching the full and partial demonstrations were not more successful in solving the task than horses receiving no human demonstration. Horses that were unsuccessful showed more human- and box-oriented behavior than successful horses, which can indicate motivation to solve the task and/or frustration from being unable to solve the task. Our study suggest that the horses did not benefit from human demonstration of how to open a box to find food.

**Abstract:**

Animals can acquire new behavior through both individual and social learning. Several studies have investigated horses’ ability to utilize inter-species (human demonstrator) social learning with conflicting results. In this study, we repeat a previous study, which found that horses had the ability to learn from observing humans performing an instrumental task, but we include a control for stimulus enhancement. One human demonstrator and thirty horses were included, and the horses were randomly assigned to one of three treatments: (A) full human demonstration, (B) partial human demonstration, and (C) no human demonstration. The task was for the horses to touch an object situated 1 m away from a feed box, to open this feed box, and thereby obtain a food reward. The success of each horse, the behavior directed towards the apparatus and the human, and behaviors indicative of frustration were observed. The results showed that horses observing a full and partial human demonstration were not more successful in solving the instrumental task than horses not observing any demonstration. Horses that did not solve the task expressed more box- and human-oriented behavior compared to successful horses, which may be an indication of motivation to solve the task and/or frustration from being unable to solve the task.

## 1. Introduction

Learning is the mechanism that changes behavior following a new experience [1] and animals can acquire new behavior through both individual and social learning. Individual learning refers to individual trial-and-error learning [2], whereas social learning has been used in the scientific literature to cover a wide range of learning mechanisms, ranging from simple processes to more cognitively complex processes [3]. As suggested by Rørvang et al. [4], we distinguish between social transmission (social facilitation, stimulus enhancement, and local enhancement, i.e., the simpler processes), and actual social learning (goal emulation and imitation). Actual social learning requires higher cognitive abilities to learn about the goal and/or actions of the demonstrator. Goal emulation reproduces the result of a demonstrator’s behavior, and copying of the exact behavior is thus not necessary as the individual understands how to achieve the result [5]. Imitation on the other hand is when the exact motor pattern of the demonstrator is copied leading the observer to perform the same behavior. It is important to distinguish between higher cognitive processes and other processes, which stand lower in cognitive complexity, as scientists should always aim for the simplest explanation of how an animal attained a particular behaviour, as formulated in Lloyd Morgan’s canon:

“In no case is an animal activity to be interpreted in terms of higher psychological processes if it can be fairly interpreted in terms of processes which stand lower in the scale of psychological evolution and development” [6].

Theoretically, social learning can happen between conspecifics (intra-species social learning) and between species (inter-species social learning). For horses, there are several studies with conflicting results both on intra- and inter-species social learning. Intra-species social learning was discussed in detail in Rørvang et al. [4], and the current study will only consider inter-species social learning. In terms of evolution, inter-species social learning is favorable when species live in overlapping niches, where information from one species can have a positive impact on the other [7], and especially so, if this information outweighs the quality of information from the same species.

Studies of inter-species social learning in horses have mainly used human demonstrators, as these are most relevant in terms of horse training. In a study by Burla et al. [8], 16 horses were challenged with a spatial detour task: locating a food reward in the middle of a simple maze. Eight horses observed a human demonstrator locating the reward, and the other eight did not. There was no effect of prior demonstration on the success rate of solving the task, nor on the latency to do so, indicating that the horses failed to learn socially from a human demonstrator. Henriksson et al. [9], tested 22 horses in a human contact-seeking experiment and a spatial detour test. The detour test was a V-shaped maze with a reward in the middle. There was no effect of prior observation of a human solving the task (i.e., following the right route to the reward) on the success rate. Interestingly, the authors found that horses expressing more task-oriented behavior also were in closer proximity to the human during the human contact-seeking experiment, and performed more eye-contact seeking behavior, but this appeared to have no effect on their ability to solve the detour task. On the contrary, Schuetz et al. [10], used an instrumental task and found a significant effect of prior human demonstration on the horses’ success rate. Although more observer horses solved the task, there was no control for local enhancement cues and the results may thus reflect social transmission of information, rather actual social learning. Moreover, the authors noted that the eight horses that solved the task initially used five different techniques to manipulate the switch, which after ten trials changed into only one technique shown by all eight horses, which further implies some level of individual learning. In a follow-up study, Bernauer et al. [11], exposed horses to different types of human demonstration, e.g., demonstrator using her hand, head, foot or hand and head for the demonstration of how to press a button to open a food box. The authors reported that more horses in the demonstration groups solved the task compared to non-demo controls, and that most horses preferred to use their head to press the button.

It is possible that humankind’s fascination with the cognitive abilities of animals can lead to conclusions that ascribe higher mental processes to animals than are actually necessary to perform a specific behavior, which is the central message of Lloyd Morgan’s canon [6]. In terms of horses, distinguishing between lower cognitive mechanisms (i.e., social transmission of information) and higher cognitive mechanisms (i.e., actual social learning) when horses observe and learn from humans is not only important from a fundamental scientific perspective. Assuming social learning mechanisms in their absence can also have implications for horse training and horse welfare [4]. If a training system assumes that horses are able to learn new behavior from observing a human performing the behavior, and the horse fails to reproduce the behavior, this can lead to frustration or even punishment. The use of punishment in training can lower the horse’s motivation to learn and reduce horse welfare [12].

The present study therefore aimed to repeat the study by Schuetz et al. [10] but including a control for local enhancement, to further investigate if horses possess the ability to utilize inter-species social learning when solving an instrumental task. This was investigated by including three treatment groups: (A) full demonstration (corresponding to the experimental group in Schuetz et al. [10]), (B) partial demonstration (controlling for local enhancement cues), and (C) no demonstration (control group as in Schuetz et al. [10]). We expected that:

(1) more horses from treatment group A and B would solve the task than from group C, if the horses rely on social transmission mechanisms (A = B > C), or that
(2) more horses from treatment group A than from B, and more horses from group B than C would solve the task, if the horses utilize actual social learning (A > B > C).

We moreover hypothesized that unsuccessful horses would express more human-oriented and frustration indicative behavior when unable to solve the task.

## 2. Materials and Methods

### 2.1. Experimental Venue and Animals

The experiment was performed at a private stud in Denmark during two consecutive weeks in March 2019. The horses (n = 30) were all Icelandic horses and either native to the stud or included into a training program at the stud. The training program consisted of basic schooling of the horses either under saddle or from the ground. The horses were a mix of mares (n = 21), stallions (n = 4), and geldings (n = 5), aged between 4 and 18 years (median age = 5.5 years, Table 1). They were pastured either during day or during night (resulting in minimum 8 h outdoors daily per horse). When not on pasture, the horses were kept in 3 × 4 m indoor, individual pens bedded with wood shavings, where they received their daily ration of feed (concentrates and hay) and waited for their training pass. The housing and management of the horses complied with national legislation. The barn with the individual pens was directly connected to an indoor riding arena where a mobile lunging circle (12 m Ø) was made from tubular metal bars (160 cm height). This lunging circle was used as the experimental venue, as all horses were familiar with being trained inside the riding arena and lunging circle. The arena ground was covered with a mixture of sand and wood shavings.

### 2.2. Experimental Design and Device

**Figure 1:**
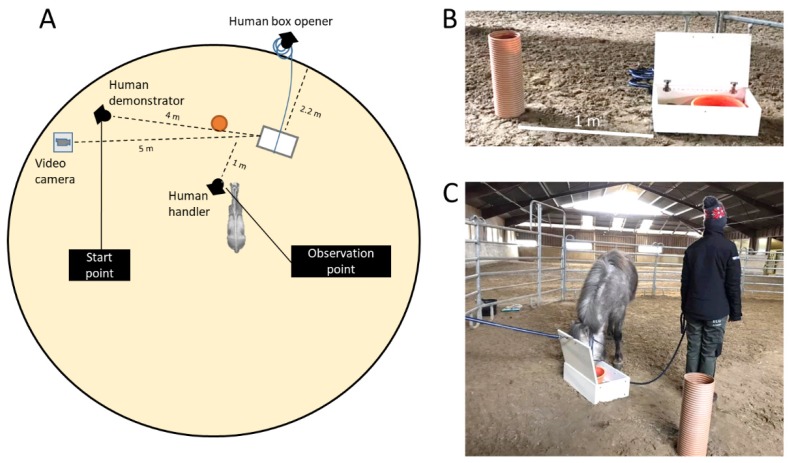
Overview of the experimental design and devices. (**A**) illustrates the top view of the lunging circle with the experimental setup. The white box illustrates the feed box, the orange circle represents the tube and the grey horse represents the proximate placement of each horse during demonstration and upon test start. Observation point and start point of demonstration is shown, as well as placement of the three persons involved in the experiment. (**B**) illustrates a close-up of the feed box and the tube. (**C**) illustrates the position of the human handler during the tests.

In the experiment, the horses were exposed to a task of opening a box containing feed by touching the top of a tube situated 1 m away from the box (adapted from [10], Video S1, Figure 1A,B). The box was 21 cm high, 63.5 cm long, and 36 cm wide, and made of wood with a white surface. The lid on top of the box was fastened with two metal hinges (on the inside of the box), and had a plastic loop on one side where a rope (8.5 m long) was fastened. A plastic bucket (33.5 cm Ø, 10 cm height) containing the feed (oat grains mixed with concentrates (pellets)) was placed in one side of the box. In the other end of the box, a large stone (~1.5 kg) was placed as a ballast weight, preventing the horses from tilting the box. The tube was made of orange, grooved, and hard plastic and measured 53 cm in height and 20 cm Ø (Figure 1B). The box and the tube were placed 1 m apart in the lunging circle facing towards an opening to the barn, which allowed visual contact with the other horses in the pens during testing. The rope attached to the box lid extended from the box to the outer side of the lunging circle (distance from box lid to fence 220 cm) whereby a person could manually open the box when a horse had performed the correct behavior (touching the tube). Five meters away from the box, a video camera on a tripod (120 cm high) recorded the experiment.

### 2.3. Experimental Procedure and Treatments

In order to investigate if horses are able to learn to solve an instrumental task from observing human demonstration, three treatments were included (n = 10 horses per treatment): (A) “Full demonstration”: the human demonstrator walked from the starting point to the tube, bent over, and touched the top of the tube with both hands for 2 sec, after which the human box opener immediately opened the box. The demonstrator then walked to the box, squatted down, and picked up feed (to approximately 20 cm above the box) and dropped the feed into the box again. (B) “Partial demonstration”: the human demonstrator walked to the tube and paused in front of the tube for 2 sec (corresponding to the duration of touching the tube in treatment (A)). The box opened as the human demonstrator paused, and the human demonstrator subsequently walked to the box, squatted down, and picked up and dropped feed as in (A). After demonstration A or B, the human demonstrator returned to the start position (Figure 1A). In the last treatment (C) “No demonstration”, no prior demonstration of the task was shown, i.e., the human demonstrator remained at the starting point. Hence, there were three persons involved: the human handler who handled all horses during habituation and testing, the human demonstrator who demonstrated the task to all horses, and, lastly, the human box opener who opened the box, ensuring that the box was always opened in the same manner.

On each experimental day, the stud owner selected a group of 3–6 same-gendered and similarly aged horses, either all native or all non-native to the stud, before the experimenters arrived at the stud. These horses were fed (at 07:00) and later trained during the first half of the day. After feeding and training, the stud owner left the horses in their respective pens in a random order. The treatment (A, B, or C) for each horse would then be blindly assigned by the experimenters following a predetermined schedule including all possible combinations of A, B, and C, which was made before arriving at the stud. In that manner, the horses were blindly allocated to the treatments in a random order, while still balancing for sex, age, and origin. The experiment was thus always performed during the second half of the day between 13:00–18:00.

### 2.4. Habituation

A habituation procedure was carried out to prepare all horses for the experimental set-up. The horses were made familiar with the human handler who led all horses during the habituation process and during testing. The horses were led individually by the human handler from their pen into the lunging circle with the experimental set-up. The handler led the horse one round in the lunging circle, which the horse was already familiar with, and then towards the open box with feed. The horse was allowed to voluntarily approach the box. If the horse walked directly to the box, it was allowed to eat three mouthfuls of the feed. If the horse showed neophobic reactions towards the box, the handler enticed it to move further by pulling lightly on the lead rope and allowing it to sniff a handful of the feed. When the horse moved closer to the box, it was rewarded a handful of feed for every step in the right direction, and finally also three mouthfuls when reaching the box. Lastly, the handler led the horse another round in the lunging circle and towards the box, allowing it to feed once again when reaching it. At this stage, the handler would turn her back towards the box upon reaching it (Figure 1C). The habituation criterion was that the horse should voluntarily approach the box and eat from it, without showing any signs of fear reactions, two times in a row. The majority of the horses needed two habituation trials (the minimum) to comply with the habituation criterion (n = 20). Ten horses needed three or four trials before complying. The duration of the habituation was on average 16 min per horse (range: 5–28 min). After complying with the habituation criterion, the horse was led back to its pen and the next horse was led into the test arena for habituation.

### 2.5. Experimental Procedure

In all but two cases, the habituation and the experimental procedure were carried out on the same day. These two horses were habituated the day before they participated in the experimental procedure (treatment group A and B).

Each horse was individually led into the lunging circle from its pen by the handler. To ensure that all horses still complied with the habituation criterion, they were first allowed to approach the open box again and feed from it as during the habituation (Figure 1C). All horses met the habituation criterion again at this stage. Hereafter, the handler led the horse away from the box and, while the horse had its hind towards the box, the human demonstrator closed the lid to the box. The horse was then led to the observation point (Figure 1A) and, depending on the assigned treatment, was either shown (A) Full demonstration, (B) Partial demonstration, or (C) No demonstration, before being allowed to try to solve the task. All horses were held on a tight rope during the demonstration, and when allowed to solve the task, the rope was loosened and the handler turned her back to the box and tube. The horse had 1 min to solve the task, after which it was led away from the box to prepare for another demonstration, and another 1 min trial. Each trial was limited to 1 min to reduce the possibility for individual learning, and each horse had a maximum of 15 trials. The experiment would thus be terminated after 15 unsuccessful trials or when the horse complied with the learning criterion. The learning criterion was defined as when the horse solved the task 10 times in a row; 5 times following demonstration (A or B) and 5 times without any demonstration. Horses in treatment C had to solve the task 10 times in a row without any demonstrations. The task was only considered successfully solved when the horse opened the box via touching the top of the tube (with the muzzle). Kicking, biting, tilting, or in other ways manipulating the box to open it was not considered successful. Such behavior (shown by two horses) was prevented by the handler pulling the rope lightly with an immediate release when the behavior stopped. Not having to prevent this behavior would have been favorable, to ensure all horses were allowed to express their preferred problem-solving behavior. Unfortunately, the box was not sufficiently durable and we thus had to prevent certain behavior. If a horse, e.g., solved the task on the 15th trial, it would be allowed additional trials in order to comply with the learning criterion. If the horse however failed on one of these additional trials before reaching the learning criterion, the experiment would be terminated. In that way, horses would receive a minimum of 10 trials (all successful trials), and could maximum receive 24 trials to comply with the learning criterion. If the horse showed no interest in the box or the tube (no approaching of the two) during two subsequent trials, the horse was given a motivation trial corresponding to a habituation trial (i.e., one round in the lunging circle and feeding from the open box) in order to regain motivation to engage in the task.

### 2.6. Behavioral Observations

From the video recordings, an observer later noted the behavior of the horses during the 1-min trials. The main response variable was whether the horses complied with the learning criterion or not (binary variable: yes/no). In addition to this variable, the number of trials before each horse successfully solved the task for the first time was noted as well as the total number of trials in order to comply with the learning criterion for each successful horse. During each trial, whether or not the horse touched the tube or the box first was noted. All box-oriented behavior (sniffing, licking, biting, pushing, and kicking the box) and human-oriented behavior (looking towards the handler, and touching the handler) was recorded as durations. Additionally, all behavior not directly related to either the handler or solving the task was noted (scratching body, head shaking, stamping, backing, and pawing > 0.5 m away from the box), as potential indicators of frustration (modified from Rochais et al. [13]). The duration of these behaviors was calculated as the mean duration per horse per trial.

### 2.7. Statistical Analyses

As none of the data was normally distributed (Shapiro-Wilk test), all analyses were non-parametrical. To investigate the effect of treatment (factorial variable with three levels: A/B/C) on number of successful horses, a χ^2^ –test was used, and when analyzing the effect of treatment on the number of trials to accomplish the learning criterion and the behaviors, Kruskal-Wallis tests were used. To investigate the effect of horses being either successful in accomplishing the learning criterion or not (factorial variable with two levels: success/not), on their expression of behavior, Mann-Whitney U test was used. When analyzing if sex (mares vs. geldings and stallions grouped) or age (young 4–6, old 7–18) affected if the horses succeeded in accomplishing the learning criterion, χ^2^ –tests were used.

All statistical analyses were performed in R version 3.6.0. “planting a tree” [14], using the core interface of the program. Graphs were produced using R package “ggplot2” [15], and subsequently refined using Microsoft Power Point 2016.

### 2.8. Ethical Statement

The owner of the stud was informed and agreed to all experimental procedures, data collection and publication. All procedures were conducted in accordance with national legislation on animal experimentation by the Danish Ministry of Justice, Act. no. nr. 253 (8 March 2013) and § 12 in Act. no. 1459 (17 December 2013), and by the Swedish Ministry of Agriculture saknr. L 150, § 18 (29 March 2019) [16] as well as met the ARRIVE guidelines [17] and the ethical guidelines proposed by the Ethical Committee of the ISAE (International Society of Applied Ethology) [18].

## 3. Results

### 3.1. Learning Criterion and Effect of Treatments

Overall, 16 out of 30 horses complied with the learning criterion, and did so using on average 15.3 ± 4.5 trials (range: 10–24, with two horses having only successful trials, and one horse having 14 unsuccessful trials). Eleven horses received 1–3 motivation trials during the experiment, two from group A, five from group B, and four from group C. Of these horses, three (one from each treatment group) ended up complying with the learning criterion. Of the 14 unsuccessful horses, five managed to solve the task once or twice (two of five). In most trials, the horses touched the top of the box before touching the tube. This behavior changed for most of the horses that successfully solved the task, after (median) four trials (range: 2–10).

There was no effect of treatment on the number of successful horses (χ^2^ –test: χ^2^ = 1.07, df = 2, *p* = 0.60, Table 2), or on the number of trials to solve the task the first time (Kruskal-Wallis test: H = 1.93, df = 2, *p* = 0.38), or on the number of trials needed to comply with the learning criterion for the successful horses (Kruskal-Wallis test: H = 1.20, df = 2, *p* = 0.55). Neither the sex nor the age of the horses had any effect on whether they complied with the learning criterion (χ^2^ –tests: χ^2^_sex_ = 1.08, df = 1, *p* = 0.30, χ^2^_age_ = 8.19, df = 7, *p* = 0.32, respectively). In addition, there was no effect of the number of habituation trials (two vs. three or four trials), on the success of the horses (χ^2^–tests: χ^2^_habituation trials_ = 1.13, df = 1, *p* = 0.22).

### 3.2. Box-Oriented Behaviour

All horses displayed box-oriented behavior (duration per horse per trial across treatments (median (25; 75% quantiles): 21.0 (12.2; 40.1)). The behavior was shown in 86% of all trials, and was not restricted to any particular trial number (1–24). Looking into the five categories of box-oriented behavior, sniffing and licking the box was most common and displayed by all horses (Table 3).

**Figure 2:**
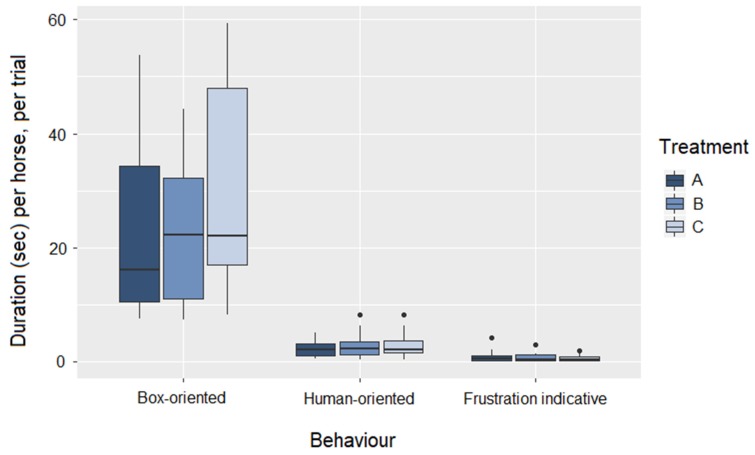
Boxplot (median, lower and upper quartiles, and min and max) of the duration of box-oriented, human-oriented and individual behavior per horse, per trial, shown by treatment A (dark blue), B (medium blue), and C (light blue).

**Figure 3:**
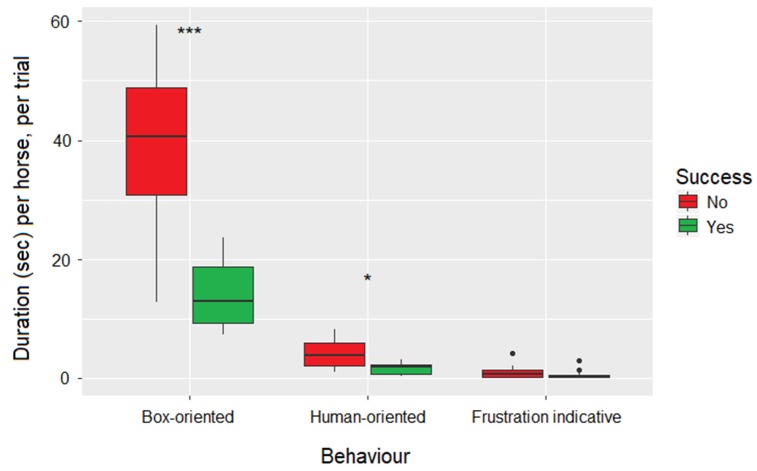
Boxplot (median, lower and upper quartiles, and min and max) of the duration of box-oriented, human-oriented, and individual behavior per horse per trial, shown by success of accomplishing the learning criterion or not (green and red, respectively). Significant differences are marked by * (*p* < 0.05), ** (*p* < 0.01) and *** (*p* < 0.001).

As the duration of the five types of box-oriented behavior was relatively low and the variance high, the five categories were summed to form one variable: total box-oriented behavior per trial (Table 3). There was no effect of treatment on the expression of total box-oriented behavior (Kruskal-Wallis test: H = 1.40, df = 2, *p* = 0.50, Figure 2). However, unsuccessful horses displayed significantly more box-oriented behavior per trial (Mann-Whitney U test, W = 208, *p* < 0.0001, Figure 3), despite having approximately the same number of trials as successful horses (unsuccessful horses 15.0 trials vs. successful horses 15.3).

### 3.3. Human-Oriented Behaviour

All but one horse expressed human-oriented behavior during their trials (summed duration per horse per trial (median (25; 75% quantiles): 2.3 (1.0; 3.6)). Human-oriented behavior was not expressed by any horses during the first or second trial. Also for this behavior, the duration was low and the variance was high, and thus the variables; look towards human handler and touch human handler were summed to total human-oriented behavior. There was no effect of treatment on the expression of human-oriented behavior (Kruskal-Wallis test: H = 0.21, df = 2, *p* = 0.90, Figure 2), but unsuccessful horses expressed significantly more human-oriented behavior during their trials (Mann-Whitney U test: W = 182, *p* = 0.004, Figure 3).

### 3.4. Frustration Indicative Behaviour (Behavior not Related to the Task)

All behavior indicative of frustration was summed due to the low duration and large variation (summed duration per horse per trial (mean ± sd): 0.8 ± 0.9). Almost all horses displayed some behavior indicative of frustration during their trials (26 out of 30), but there was no effect of treatment on this expression (Kruskal-Wallis test: H = 0.19, *p* = 0.91, Figure 2). The successful and unsuccessful horses did not differ in the expression of this behavior (Mann-Whitney U test: W = 147.5, *p* = 0.15, Figure 3). Interestingly, these behaviors were never expressed during the first three trials and were most prevalent during unsuccessful trials succeeding a successful trial (11 out of 14 unsuccessful trials following a successful).

## 4. Discussion

This study investigated the effect of three different types of human demonstration on horses’ ability to solve an instrumental task: full, partial, and no demonstration. The results showed no significant effect of prior demonstration on the number of successful horses. The number of trials needed to accomplish the first opening of the box and to comply with the learning criterion were also unaffected by demonstration, contradicting our expectations about social transmission or social learning, as described in the introduction:

(1) more horses from treatment group A and B would solve the task than from group C, if the horses rely on social transmission mechanisms (A = B > C), or that
(2) more horses from treatment group A than from B, and more horses from group B than C would solve the task, if the horses utilize actual social learning (A > B > C).

The expression of the observed behaviors (human-oriented behavior, box-oriented behavior, and behavior potentially indicative of frustration) also appeared unaffected by demonstration, but unsuccessful horses expressed more human-oriented and box-oriented behavior than successful horses. Potential frustration behavior was not affected by demonstration or if the horses were successful or not, but was almost exclusively expressed during an unsuccessful trial succeeding a successful one.

These results contradict the findings by Schuetz et al. [10] and Bernauer et al. [11] where it was reported that horses observing a human demonstration were more successful in opening a box by pressing a switch compared to horses not observing a demonstration. The different results could be a result of differences in the experimental designs as Schuetz et al. [10] and Bernauer et al. [11] used a mechanical switch, whereas in our study the horses touched the top of a tube. It could be that touching the top of a tube is easier than pushing a switch as the switch is smaller and may require more force to activate the opening of the feed box. Thus, the task in our study may not require demonstration. However, 67% and 75% of the horses that observed a human demonstration solved the task in Schuetz et al. [10] and Bernauer et al. [11], respectively, whereas only 60% of the horses solved the task in the current experiment, following full and partial human demonstration, suggesting that the learning criterion was not more easily achieved in the present study. Another difference was the maximum number of demonstrations which was 120 in Schuetz et al. [10] and Bernauer et al. [11] and only 15 in the present study. The horses in our experiment achieved the learning criterion after 15.3 trials on average, compared to 17.5 on average in Schuetz et al. [10], suggesting that the high number of demonstrations in the previous studies did not enhance the chance of accomplishment of the task. Although four horses from our control group managed to solve the task without any demonstration, 9 out of 14 unsuccessful horses did not solve the task using the correct behavior even once (N: A = 2, B = 3, C = 4), implying that the task was challenging enough for 40% of the horses not to succeed at all.

In the present study, horses failed to learn from observing humans in an instrumental task. This finding corresponds with the recent results from Burla et al. [8] and Henriksson et al. [9], who also found no effect of human demonstration on horses’ success rate in a detour task, and moreover with the majority of the studies on intra-species social learning in horses (for a general review: Rørvang et al. [4]). For example, Lindberg et al. [19] and Ahrendt et al. [20] found no effect of prior demonstration from a conspecific on the number of observer horses accomplishing an instrumental task, nor on their latency to do so. Ahrendt et al. [20] and Henriksson et al. [9] however found that successful horses expressed more device/task-oriented behavior after demonstration, which implies that the successful horses might have used social facilitation or local enhancement cues from the demonstrator, making them more likely to engage in device or task-oriented behavior. Our study did not confirm this finding, as there was no effect of demonstration on the expression of box-oriented behavior, but instead we found that unsuccessful horses expressed more box-oriented behavior. We defined box-oriented behavior as physical contact with the box or placing the head less than 10 cm from the box (corresponding to the length of a muzzle), which is likely linked to attempts of solving the task. In addition, unsuccessful horses also expressed more human-oriented behavior adding more support to the frustration hypothesis. It is possible that unsuccessful horses may have expected an action from the human situated close to them [21,22]. Proops et al. [23] suggested that horses adjust their begging behavior to human attentive states and are sensitive to human gaze. We tried to avoid the effect of human gaze by having the handler turn her back to the horse during testing, but the horse may still have sought her attention in order to solve the instrumental task. The finding that unsuccessful horses expressed more human-oriented behavior is in accordance with Schuetz et al. [10] and Bernauer et al. [11], but we found no effect of demonstration on this behavior, in contrast to Schuetz et al. [10]. Horses have been reported to use human local enhancement cues when solving a three-way object-choice task [24], and to use human pointing gestures when locating food [25], making the lacking effect of human demonstration somewhat disappointing. On the other hand, the human local enhancement cues (pointing or gazing) in these two studies were given while the horse was choosing, which inevitably makes the task easier as compared to remembering the pointing or gazing gesture and then making a choice. As horses did not seem to utilize information from the human demonstration in our studies, they may have been more attentive towards the human in the near proximity. Krueger et al. [24] reported that horses tend to choose the baited container situated next to a human irrespective of the persons attentive state. In that way, the unsuccessful horses may have used the human handler as a local enhancement cue when being unable to solve the task immediately, which however did not increase their chance of solving the task. It therefore remains unclear if horses can utilize socially transmitted cues from humans when solving instrumental tasks.

## 5. Conclusions

This small scale study found that horses observing a full or partial human demonstration were not more successful in solving an instrumental task, than horses that did not observe any demonstration. Thus, in practical horse training, one should not expect horses to be readily able to solve an instrumental task, such as opening a feed box, following demonstration by a human. Unsuccessful horses expressed more box- and human-oriented behavior than successful horses. The human-oriented behavior expressed by unsuccessful horses may reflect them seeking human assistance as also suggested in previous studies, but this would need to be confirmed in a larger study.

## Figures and Tables

**Table 1:** Overview of gender and age distribution within treatment groups A, B, and C.

	Treatment Group A	Treatment Group B	Treatment Group C
Stallions	1	2	1
Geldings	1	2	2
Mares	8	6	7
Age (median (range))	5 (4–7)	6 (5–10)	5 (5–18)

**Table 2:** Overview of the treatments groups, the successful horses in each group, the number of trials before the first opening of the box (1st success) and to accomplish the learning criterion, and total trials (median: 25;75% quantiles).

Treatment	N_total_	No. Successful Horses	Trials to 1st Success	Trials to Accomplish Learning Criterion for Successful Horses
A (Full demo)	10	6	2 (2;4.8)	13.5 (11.5;14.8)
B (Partial demo)	10	6	4 (3;6)	15.5 (13;21)
C (No demo)	10	4	5.5 (3.5;9)	15.5 (13;18.8)

**Table 3:** Overview of box-oriented behaviors presented as the (median (25; 75% quantiles)) duration per horse in seconds.

Treatment	Pushing Box	Sniffing Box	Licking Box	Biting Box	Kicking Box	Total Box-Oriented
A	1.0 (0.0; 12.5)	105 (73.5; 137.5)	68.0 (32.0; 188.0)	3.0 (0.0; 6.0)	2.5 (1.0; 4.8)	16.2 (10.4; 34.4)
B	1.0 (0.0; 1.0)	90.0 (72.8; 147.0)	188.0 (75.0; 272.0)	7.0 (0.5; 14.0)	1.0 (1.0; 3.5)	22.2 (11.0; 32.2)
C	0.0 (0.0; 1.0)	106.5 (84.5; 175.5)	164.0 (74.0; 294.0)	4.0 (0.0; 11.0)	2.5 (2.0; 3.0)	22.2 (17.0; 48.0)

## Supplementary Materials

The following are available online at https://www.mdpi.com/2076-2615/10/2/221/s1, Video S1: Exemplified illustration of the feed box, the tube and how a successful trial was performed by a horse (this particular horse had already finished testing).
